# Evaluation of an adapted relative growth method by MALDI-TOF MS for rapid determination of the susceptibility of *Escherichia coli* to levofloxacin

**DOI:** 10.1128/spectrum.01035-26

**Published:** 2026-08-04

**Authors:** Yulong Liu, Niqi Xie, Weiwei Hu, Xiaoqin Zeng, Siying Sun, Lijuan Pan, Hongyou Chen, Xuelian Peng, Chunyan Yang, Baoru Han, Jin Li

**Affiliations:** 1 Department of Laboratory Medicine, The Affiliated Dazu’s Hospital of Chongqing Medical University, Chongqing, China; 2 College of Artificial Intelligence Medicine, Chongqing Medical University 12550, Chongqing, China; MultiCare Health System, Tacoma, Washington, USA

**Keywords:** antimicrobial susceptibility testing, MALDI-TOF MS, *Escherichia coli*, levofloxacin, adapted relative growth method, AI MedLab MS

## Abstract

Antimicrobial resistance (AMR) poses a critical global health threat. Conventional antimicrobial susceptibility testing (AST) requires 12–24 h, delaying targeted treatment. Although rapid AST approaches exist, their reliance on static measurements and single-colony analysis limits diagnostic accuracy by overlooking intrasample heterogeneity, which can produce misleading results when resistant subpopulations are present. Here, we present a novel analytical framework that integrates dynamic MALDI-TOF MS spectral analysis, the robust dynamic relative growth (RBD-RG) algorithm, and machine learning. This framework decodes the mass spectral evolution of *Escherichia coli* exposed to levofloxacin, enabling the rapid determination of levofloxacin susceptibility. AI MedLab MS was developed and validated using 60 clinical *E. coli* isolates, achieving robust classification performance across resistance phenotypes. Prospective validation with 50 independent isolates confirmed clinical utility: AI MedLab MS delivered AST results within 2 h, with an overall accuracy of 84.0% and a precision of 95.8% for resistant isolates. By shifting the analytical paradigm from single-timepoint detection to dynamic trajectory modeling, this study demonstrates a precise and interpretable methodology for rapid AST. The platform addresses key limitations of conventional methods by analyzing the overall bacterial population rather than individual colonies, thereby improving classification accuracy for complex resistance phenotypes, and has the potential to provide actionable clinical decision-making within a 2-h window.

**IMPORTANCE:**

Rapid determination of antimicrobial susceptibility is critical for the management of severe bacterial infections, yet conventional culture-based assays require up to 24 h. While emerging rapid diagnostic tools offer shorter turnaround times, their accuracy is frequently limited by a reliance on static phenotypic endpoint measurements. Here, we report AI MedLab MS, a machine-learning-enabled diagnostic platform that captures the real-time, dynamic response profiles of *Escherichia coli* exposed to levofloxacin. By longitudinally tracking these continuous phenotypic transitions, our method differentiates drug-resistant strains within 2 h. This rapid profiling capability may provide actionable diagnostic insights to guide targeted therapy, with the potential to optimize clinical decision-making and support antimicrobial stewardship.

## INTRODUCTION

Antimicrobial resistance (AMR) has emerged as a major global public health challenge, with its increasing prevalence posing a serious threat to the efficacy of clinical anti-infective therapies (1–3). *Escherichia coli*, an important component of normal human intestinal flora, is among the most common opportunistic pathogens in clinical practice (4, 5). It is associated with a range of infections, including urinary tract infections (UTIs), bloodstream infections, and intra-abdominal infections, thereby making it a significant pathogen in both community-acquired and hospital-acquired settings (6, 7). Fluoroquinolones are among the most widely prescribed antibiotic classes globally, with consumption data showing their extensive use across both high-income and low- and middle-income countries (8). Levofloxacin (LEV), a broad-spectrum fluoroquinolone, is a first-line agent for the treatment of urinary tract infections (UTIs) and systemic infections caused by *E. coli* owing to its potent inhibition of bacterial DNA gyrase and topoisomerase IV (9, 10). However, the global expansion of fluoroquinolone use has been accompanied by a steady increase in LEV resistance among *E. coli* isolates in recent years (11, 12). This alarming trend not only increases the risk of treatment failure but also significantly complicates the formulation of clinical anti-infective regimens. Consequently, rapid and reliable AMR detection technologies in clinical microbiology laboratories are urgently needed.

Current antimicrobial susceptibility testing (AST) methods, notably the Clinical and Laboratory Standards Institute (CLSI) broth microdilution method, remain the gold standard for clinical resistance detection (13). Automated commercial systems such as the VITEK 2 Compact (BioMérieux, France) are widely used as comparator methods, with instrument turnaround times of approximately 12–18 h for *Enterobacterales* (14–16). The overall workflow from positive blood culture, including isolation, identification, and AST, typically requires 48–96 h (17). To shorten this interval, several rapid direct-from-blood-culture phenotypic AST methods have been developed, including the Accelerate Pheno system (approximately 7 h) (18) and the VITEK REVEAL system (approximately 5 h) (19). Molecular techniques, such as reverse transcription polymerase chain reaction (RT-PCR) and multiplex molecular panels (e.g., the BioFire FilmArray Blood Culture Panel), can further shorten detection time to several hours by targeting and amplifying known resistance genes (20–22). However, these approaches detect gene presence rather than active expression and cannot confirm whether identified resistance determinants are conferring phenotypic resistance. Moreover, their widespread clinical adoption remains hindered by high costs and incomplete coverage of resistance determinants.

Matrix-assisted laser desorption/ionization time-of-flight mass spectrometry (MALDI-TOF MS) has evolved into a core technology for clinical microbial identification because of its speed, cost-effectiveness, and high throughput (23, 24). In recent years, its application has been extended to the detection of AMR. Among the proposed approaches, the MALDI Biotyper antibiotic susceptibility test rapid assay (MBT-ASTRA) introduced by Lange et al. (25, 26) is particularly notable. This method is used to enable resistance determination within 2–4 h by comparing the mass spectral profiles of bacteria following short-term incubation in drug-containing and drug-free media. Despite this progress, MBT-ASTRA still suffers from procedural complexity and limited result stability. These limitations arise primarily from the reliance on a single measurement during the transient phase of the bacterium–antibiotic interaction. Unlike reference methods such as broth microdilution, which assess growth once the system has reached equilibrium, MBT-ASTRA captures only a snapshot of a dynamic process and therefore cannot reliably predict the final resistance phenotype (27, 28).

Artificial intelligence (AI) technology offers a promising avenue for overcoming these bottlenecks. Unlike traditional statistical approaches that require predefined feature selection and assumptions about variable relationships, machine learning methods can identify complex nonlinear patterns directly from high-dimensional spectral data (27, 29). A key advantage of AI models lies in their ability to automatically extract dynamic patterns that are indiscernible to human operators, thereby enabling standardized, automated data analysis and decision-making. To address the clinical demand for rapid, accurate, and user-friendly AST, this study integrates AI MedLab MS, an automated system that integrates the adapted relative growth method with dynamic MALDI-TOF MS and AI, for the rapid determination of the susceptibility of *E. coli* to LEV.

## MATERIALS AND METHODS

### Study design and clinical cohort

**Fig 1:**
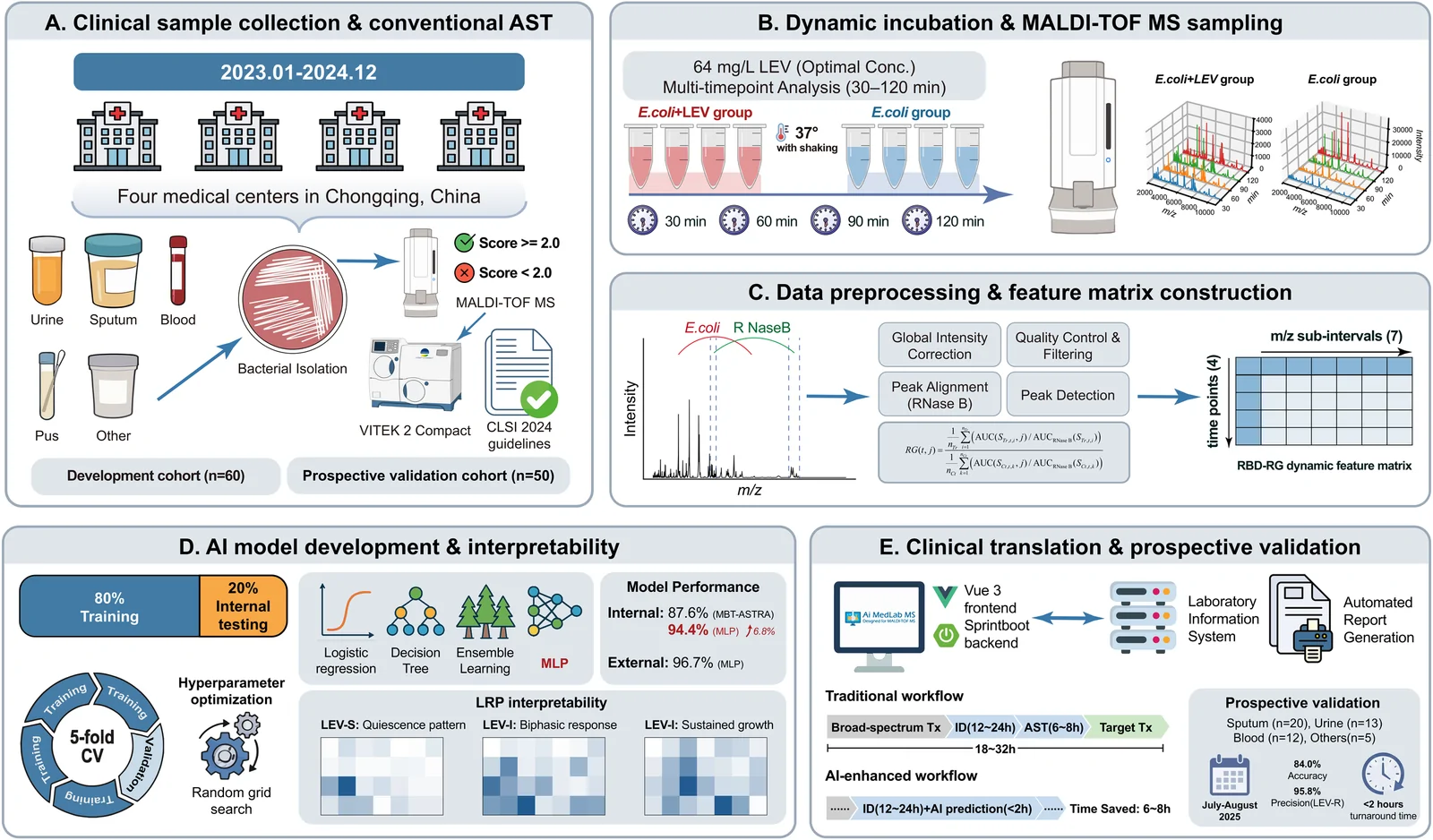
Integrated workflow of the AI MedLab MS platform for rapid resistance prediction via dynamic spectral profiling. (**A**) Clinical sample collection and conventional AST. Clinical *E. coli* isolates were collected from four medical centers in Chongqing (January 2023–December 2024). Specimens included urine, sputum, blood, pus, and others. Following bacterial isolation, species identification (MALDI-TOF score ≥ 2.0) and comparator AST (VITEK 2 Compact, CLSI 2024) were performed to establish the ground truth for the development cohort (*n* = 60) and prospective validation cohort (*n* = 50). (**B**) Dynamic incubation and MALDI-TOF MS sampling. Bacterial suspensions were incubated under an optimized condition (64 mg/L LEV) at 37°C with shaking. Parallel multi-timepoint analysis was conducted at 30, 60, 90, and 120 min. The 3D spectral plots illustrate the kinetic evolution of signal intensities over time for the treated (*E. coli* + LEV) vs control (*E. coli*) groups. (**C**) Data preprocessing and feature matrix construction. The raw spectral processing pipeline involves four key steps: Global Intensity Correction, Quality Control & Filtering, Peak Alignment using RNase B, and Peak Detection. These steps feed into the RBD-RG calculation (formula shown), generating a structured RBD-RG dynamic feature matrix (7 subintervals × 4 time points). (**D**) AI model development and interpretability. The development workflow utilized an 80% training/20% internal testing split with fivefold cross-validation (CV) and random grid search. Four model types were evaluated: Logistic Regression, Decision Tree, Ensemble Learning, and MLP. The MLP model achieved 94.4% accuracy, outperforming the conventional MBT-ASTRA method (87.6%). LRP interpretability visualizes phenotype-specific patterns: quiescence (LEV-S), biphasic response (LEV-I), and sustained growth (LEV-R). (**E**) Clinical translation and prospective validation. The system architecture integrates a Vue 3 frontend and Spring Boot backend, designed to interface with the LIS for automated reporting. The AI-enhanced workflow reduces the diagnostic timeline, saving 6–8 h compared to the traditional workflow. The prospective validation (July–August 2025) on diverse samples (Sputum *n* = 20, Urine *n* = 13, Blood *n* = 12, and Others *n* = 5) confirmed an accuracy of 84.0% and a precision of 95.8% for LEV-R with a <2-h turnaround time.

This multicenter study utilized clinical *E. coli* isolates obtained from four medical institutions in Chongqing, China: the Affiliated Dazu’s Hospital of Chongqing Medical University, the First Affiliated Hospital of Chongqing Medical University, the Chenjia Bridge Hospital of Chongqing Medical and Pharmaceutical College, and the Daping Hospital of the Army Medical University. To ensure rigorous model development and evaluation, the study design comprised two distinct phases utilizing independent data sets. The retrospective development cohort consisted of 60 independent isolates collected between January 2023 and December 2024, which were purposefully selected to ensure a balanced distribution of phenotypes (Fig. 1A). To evaluate the geographic generalizability of the model, this cohort was stratified by collection site: isolates from three centers served as the training and internal testing set, while isolates from the fourth center constituted an independent external validation set (Fig. S1A). To determine the platform’s utility in a real-world clinical workflow, a prospective validation cohort comprising 50 consecutive, independent isolates was subsequently enrolled at the Affiliated Dazu’s Hospital of Chongqing Medical University between July and August 2025. These isolates were derived from a diverse range of clinical samples, including urine, sputum, peripheral blood, wound exudate, and other body fluids. To maintain protocol standardization, all specimens were centrally processed at the clinical microbiology laboratory of the Affiliated Dazu’s Hospital of Chongqing Medical University.

### Bacterial culture, identification, and antimicrobial susceptibility testing

Isolates were subcultured on Columbia blood agar plates (Oxoid, UK) and incubated overnight at 37°C with 5% CO_2_. Species identification was performed using the EX-Accuspec MALDI-TOF MS system (Zybio, China, Version 3.3.4.8). For sample preparation, fresh single colonies were picked and smeared onto the target plate to form a thin film. Following air-drying, the spots were overlaid with 1 µL of 70% formic acid. Once dried, 1 µL of HCCA matrix solution was added and allowed to crystallize at room temperature. Identification confidence scores ≥2.0 were considered reliable for inclusion. Antimicrobial susceptibility testing was performed using the VITEK 2 Compact system (BioMérieux, France) with AST-N334 cards and VITEK 2 Systems Release version 9 software. The VITEK 2 Compact system performs automated AST based on broth microdilution principles and uses CLSI-derived interpretive criteria. Isolates were phenotypically classified as levofloxacin-susceptible (LEV-S), levofloxacin-intermediate (LEV-I), or levofloxacin-resistant (LEV-R) in accordance with the 2024 CLSI guidelines (Fig. S1B). All experimental procedures were conducted in a biosafety level 2 (BSL-2) laboratory.

### Sample preparation and dynamic spectral profiling

A preliminary dose-finding experiment tested five LEV concentrations (8, 16, 32, 64, and 128 mg/L), covering the CLSI 2024 susceptible (≤0.5 mg/L) and resistant (≥2 mg/L) breakpoints for LEV against *E. coli*. A concentration of 64 mg/L was selected for subsequent dynamic profiling, as it produced the most discriminative spectral differences between susceptible and resistant isolates within the 2-h observation window. The dynamic profiling procedure was as follows: a single well-isolated colony was picked from a Mueller-Hinton (MH) agar plate with a sterile inoculation loop and suspended in Brain Heart Infusion (BHI) broth to prepare a 0.5 McFarland standard suspension. The suspension was divided into two equal aliquots. One aliquot was supplemented with a defined concentration of LEV; the other served as an untreated blank control. Strict operational protocols were followed throughout the experiment to avoid cross-contamination.

The two suspensions were incubated at 37°C with shaking, and samples were taken at four time points (500 µL every 30 min). Collected samples were transferred to centrifuge tubes and centrifuged at 13,000 rpm (17,390 × *g*) for 2 min at room temperature. The supernatant was discarded, and the pellet was washed with 100 µL of sterile water, followed by a second centrifugation. Subsequently, 100 µL of 75% ethanol was added, followed by gentle shaking and centrifugation under the same conditions. The pellet was then air-dried. Afterward, 10 µL of 70% formic acid was added, mixed thoroughly, and incubated at room temperature for 2 min to disrupt the bacterial cell wall and release intracellular components. Subsequently, 10 µL of 100% acetonitrile containing an internal standard (RNase B, 20 g/L; Sigma-Aldrich) was added, followed by shaking and centrifugation. A 1 µL sample was then applied to the target plate and air-dried, and 1 µL of HCCA matrix solution (α-cyano-4-hydroxycinnamic acid; Zybio, China) was added and air-dried (Fig. S2). The prepared target plate was analyzed using an EX-Accuspec mass spectrometer in linear positive ionization mode. Instrument parameters included an accelerating voltage of 20 kV, a nitrogen laser frequency of 60 Hz, and an *m/z* range of 2,000–20,000 Da. Each spectrum was generated by accumulating 240 laser shots. Randomized sampling paths across six independent biological replicates per strain/condition, with measurements repeated in two independent runs, were employed for data acquisition.

### Data preprocessing and RBD-RG feature extraction

Raw mass spectral data were exported following automated baseline subtraction and smoothing using the instrument vendor software (EX-Accuspec Version 3.3.4.8). Computational processing was performed using Python 3.11 and SciPy 1.13.1. To ensure intersample comparability, global peak intensity alignment was anchored to the RNase B internal standard signals, which were specifically localized to the 7,400–7,800 *m/z* ([M + 2H]²^+^) and 14,800–15,600 *m/z* ([M + H]^+^) regions (Fig. 1C and Fig. S3 and S4). Quality control (QC) automatically filtered out low-quality spectra on the basis of predefined scoring rules targeting the internal standard peaks (Fig. S5 and S6 and Table S1).

The robust dynamic relative growth (RBD-RG) algorithm was used to quantify the temporal changes in the bacterial proteome. The informative bacterial spectral range (3,400–14,800 *m/z*) was subdivided into 11 fixed-width subintervals (1,000 *m/z* each). Subintervals overlapping with the internal standard region or exhibiting high data sparsity (containing ≥90% zero or infinite values) were excluded. This feature selection process yielded seven distinct informative subintervals (Fig. 1B).

For each valid subinterval j at time point t, the RBD-RG value represents the ratio of the normalized bacterial signal intensity in treated samples ($T_{r}$) vs control samples ($C_{r}$), which is calculated as follows:


(1) $$RG(t,j)=\frac{\frac{1}{n_{Tr}}\sum_{i=1}^{n_{Tr}}\left(\text{AUC}(S_{Tr,t,i},j)/{\text{AUC}}_{\text{RNase B}}(S_{Tr,t,i})\right)}{\frac{1}{n_{Ct}}\sum_{k=1}^{n_{Ct}}\left(\text{AUC}(S_{Ct,t,k},j)/{\text{AUC}}_{\text{RNase B}}(S_{Ct,t,k})\right)}$$

where $n_{Tr}$ and $n_{Cr}$ denote the number of replicates for treated and control groups, respectively. $\text{AUC}(S_{\cdot ,t,\cdot },j)$ is the area under the curve of bacterial peaks in subinterval j.${\text{AUC}}_{\text{RNase B}}(S_{\cdot ,t,\cdot })$ is the total AUC of the internal standard peaks in the corresponding spectrum.

This calculation yields a dynamic feature matrix for each isolate:


(2) $$M_{\text{RBD-RG}}=[RG(t,j){]}_{4\times 7}$$

Here, t∈{30,60,90,120 min} and j∈{1,…,7}. Instead of a static binary outcome, this transforms the response of each isolate into a structured spatiotemporal feature matrix. This matrix was subsequently flattened into a 28-dimensional spatiotemporal feature vector to serve as the input for machine learning models.

### Machine learning development and comparison

The data set from the three training centers was partitioned into a training set (80%) and an internal testing set (20%) using stratified random sampling to preserve the class distribution of resistance phenotypes. We systematically evaluated four classifiers to capture both linear and nonlinear data patterns: logistic regression (LR), decision tree (DT), extreme gradient boosting (XGBoost), and a multilayer perceptron (MLP) neural network. Hyperparameter optimization was conducted via a randomized grid search strategy within a fivefold cross-validation framework on the training data, utilizing the area under the receiver operating characteristic curve (AUROC) as the maximization objective (Table S2). Key classification metrics, including accuracy, precision, recall, F1-score, and AUROC, were calculated. These metrics were computed using a one-vs-all strategy, treating each class as the positive class against all others in a binary classification framework. The corresponding calculation formulas are presented as follows:


(3) $$\text{TPR}=\frac{\text{TP}}{\text{TP}+\text{FN}}$$

(4) $$\text{FPR}=\frac{\text{FP}}{\text{FP}+\text{TN}}$$

(5) $$\text{AUROC}=\int_{0}^{1}\text{TPR}(\text{threshold})\text{d}(\text{FPR}(\text{threshold}))$$

(6) $$\text{Accuracy}=\frac{\text{TP}+\text{TN}}{\text{TP}+\text{TN}+\text{FP}+\text{FN}}$$

(7) $$\text{Precision}=\frac{\text{TP}}{\text{TP}+\text{FP}}$$

(8) $$\text{Recall}=\frac{\text{TP}}{\text{TP}+\text{FN}}$$

(9) $${\text{F}}_{1}\text{-score}=2\cdot \frac{\text{Precision}\cdot \text{Recall}}{\text{Precision}+\text{Recall}}$$

where TP, TN, FP, and FN represent true positives, true negatives, false positives, and false negatives, respectively. The F1-score, which ranges from 0 to 1, provides a balanced measure of precision and recall; values above 0.80 are generally considered good, while values above 0.90 indicate excellent classification performance.

To assess the robustness of the predictive model and account for variability, all performance metrics were reported as the mean ± standard deviation (SD) derived from 10 independent run iterations.

### Interpretability analysis

To visualize the decision-making logic of the neural network, layerwise relevance propagation (LRP) was implemented (Fig. 1D). This technique propagates the prediction output backward through the network layers to assign a relevance score to each input feature (specific time point and *m/z* subinterval). By visualizing these relevance scores as heatmaps, we identified the specific spatiotemporal windows driving the classification of each resistance phenotype. All machine learning workflows were implemented using Python 3.11 with Scikit-learn 1.3.0 libraries.

### Platform deployment and clinical workflow integration

The best-performing model was deployed as the web-based AI MedLab MS platform (Fig. S7). The platform architecture features a Spring Boot backend for service orchestration and a Vue 3 frontend for the user interface. The system interfaces with the laboratory information system (LIS) to enable automated data retrieval and resistance prediction. This deployed system was utilized for the prospective validation phase.

## RESULTS

### Optimization of levofloxacin exposure conditions

**Fig 2:**
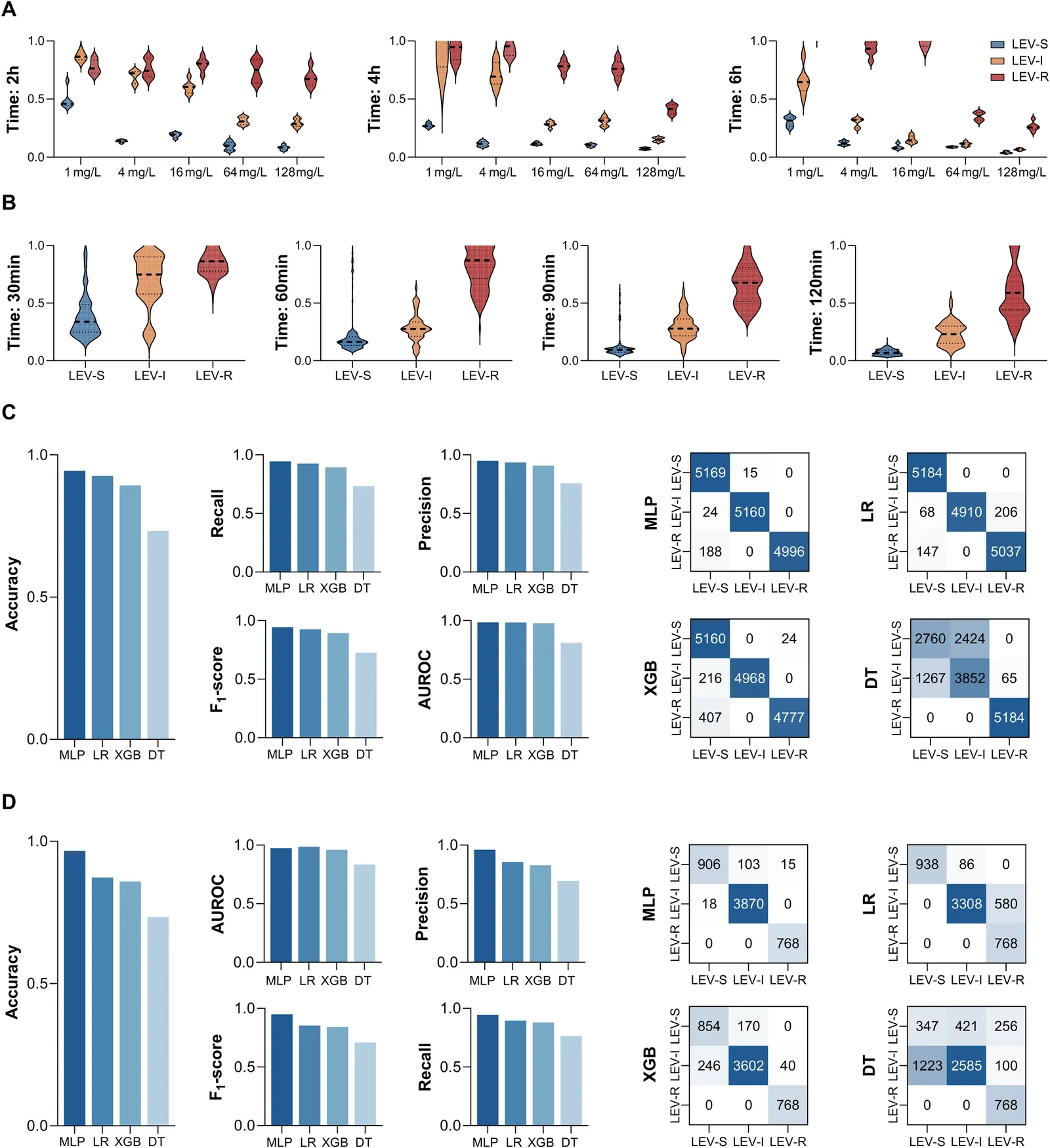
Optimization of the MBT-ASTRA method and comparative evaluation of machine learning models for AMR prediction. (**A**) Optimization of incubation time and levofloxacin (LEV) concentration. Violin plots showing RG values for levofloxacin-susceptible (LEV-S), -intermediate (LEV-I), and -resistant (LEV-R) *E. coli* strains tested at various LEV concentrations and incubation times. The condition of 64 mg/L LEV at 2 h (indicated by the dashed box) was selected for providing optimal separation. (**B**) Temporal dynamics of bacterial growth under optimized conditions. RG values for the three phenotypes monitored over 120 min under the optimized conditions, illustrating the progressive separation of growth patterns. (**C**) Performance evaluation of four machine learning models on the internal testing set. Key classification metrics and corresponding confusion matrices for four machine learning models evaluated on the internal test set. (**D**) Performance evaluation of the models on the external validation set. Performance of the models on an independent external validation set, demonstrating the superior accuracy and generalization of the MLP model.

To determine the optimal antibiotic pressure, the proteomic response of representative *E. coli* isolates was mapped across a gradient of LEV concentrations (1–256 mg/L). At lower concentrations, susceptible isolates exhibited insufficient growth inhibition, leading to overlapping trajectories with those of resistant strains. However, as the concentration increased, the divergence between phenotypes became more pronounced. Quantitative analysis indicated that 64 mg/L provided the maximum phenotypic separation (i.e., the greatest difference in RG values) between susceptible and resistant populations within the critical 2-h observation window (Fig. 2A). Consequently, this concentration was established as the standard exposure condition for all subsequent dynamic profiling assays.

### Temporal monitoring of bacterial response uncovers divergent resistance trajectories

Longitudinal monitoring over 120 min revealed that resistance phenotypes are distinguished by dynamic growth patterns, capturing temporal fluctuations that are otherwise lost in single-timepoint assessments. At the 30-min mark, RG values for LEV-R strains overlapped considerably with those for the other phenotypes, thereby limiting discriminatory power (Fig. 2B). However, as incubation progressed, a clear bifurcation in growth patterns emerged. By 120 min, distinct RG thresholds allowed for phenotype classification: LEV-S isolates exhibited pronounced inhibition (RG < 0.101), while LEV-R isolates maintained robust growth (RG > 0.396).

Crucially, LEV-I isolates fell between these distinct clusters, often displaying complex intermediate responses. Despite the optimized conditions, conventional analysis based on single-timepoint RG values achieved an overall accuracy of 87.6%. Notably, this static approach struggled to capture the complexity of the intermediate phenotype, yielding a precision of only 42.2% for LEV-I strains (Table S3).

### Machine learning enables precise classification by decoding high-dimensional spectral dynamics

In the internal testing, we systematically evaluated four machine learning algorithms. The MLP model demonstrated superior performance, achieving the highest overall accuracy of 0.944 ± 0.040 (Fig. 2C). While LR performed comparably in the internal set (accuracy: 0.926 ± 0.034), traditional tree-based models struggled to capture the complex temporal dependencies. Specifically, DT and XGBoost models yielded significantly lower accuracies of 0.733 ± 0.061 and 0.893 ± 0.073, respectively (Table S4). Crucially, the MLP model exhibited exceptional robustness in identifying the challenging intermediate phenotype, achieving an F1-score of 0.968 ± 0.029 for LEV-I strains, outperforming XGBoost (0.877 ± 0.094) and DT (0.679 ± 0.067).

The generalization capability of the platform was confirmed using an independent external validation set. In this rigorous test, the performance gap between the models widened significantly. The MLP model maintained a high accuracy of 0.967 ± 0.018, whereas the accuracy of the LR model dropped to 0.873 ± 0.052, and the DT model fell to 0.734 ± 0.094 (Table S4). This disparity highlights the distinct advantage of MLP architecture in mitigating site-specific batch effects and maintaining diagnostic stability across different medical centers.

### Interpretability analysis reveals distinct dynamic signatures underlying resistance phenotypes

**Fig 3:**
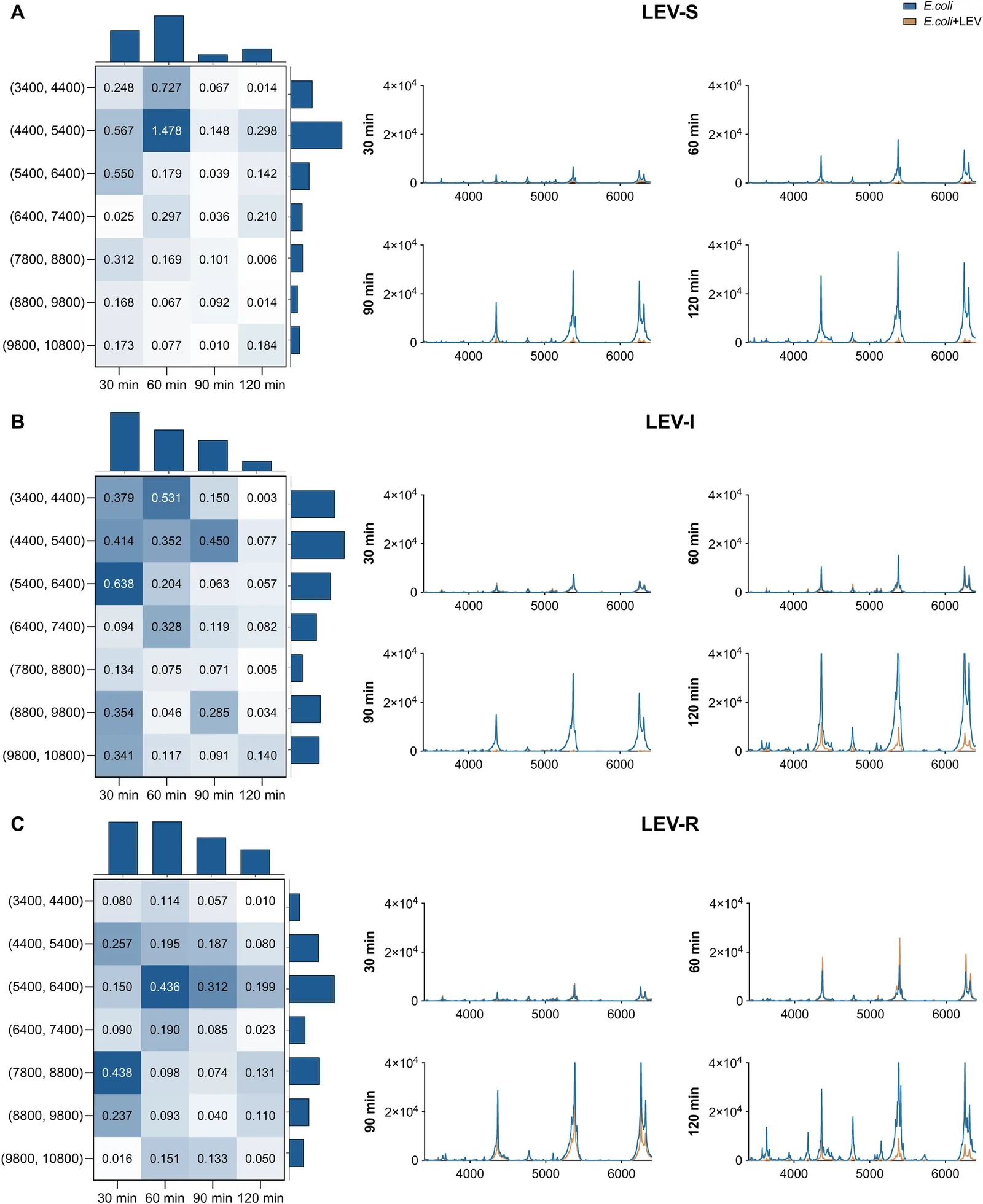
Interpretability of the MLP model using LRP reveals phenotype-specific decision patterns. For each phenotype, left panels show LRP heatmaps of feature importance across time and *m/z* subintervals. Right panels show the corresponding raw mass spectra from the most influential subintervals for drug-free control and LEV-treated conditions. (**A**) LEV-S: for susceptible isolates, the model’s decision is predicated on identifying a state of relative quiescence. The LRP heatmap displays low, diffuse relevance scores across most features, indicating that the absence of a strong, dynamic response is the key predictive signal. (**B**) LEV-I: the model’s decision is driven by a highly dynamic signature, with relevance scores peaking in the 4,400–6,400 *m/z* range at early-to-mid time points (≤90 min). The spectra reveal this corresponds to a unique biphasic response. (**C**) LEV-R: a key relevance hotspot at 60 min in the 5,400–6,400 *m/z* range is the primary determinant for resistance prediction, which matches the sustained growth observed in the spectra.

LRP was employed to visualize the decision-making logic of the MLP model. For LEV-S isolates, the model’s decision was associated with an absence of dynamic spectral changes relative to the control (Fig. 3A). Conversely, for LEV-R isolates, relevance hotspots were localized to sustained signal intensities in specific *m/z* ranges (e.g., 5,400–6,400 *m/z*) at later time points (Fig. 3C).

For the LEV-I phenotype, the model identified a distinct dynamic signature from that of both S and R. High relevance scores were concentrated in the early incubation phase (≤90 min), corresponding to a biphasic response observed in the raw spectra (Fig. 3B).

### Prospective validation confirms rapid turnaround and actionable precision in a clinical workflow

**Fig 4:**
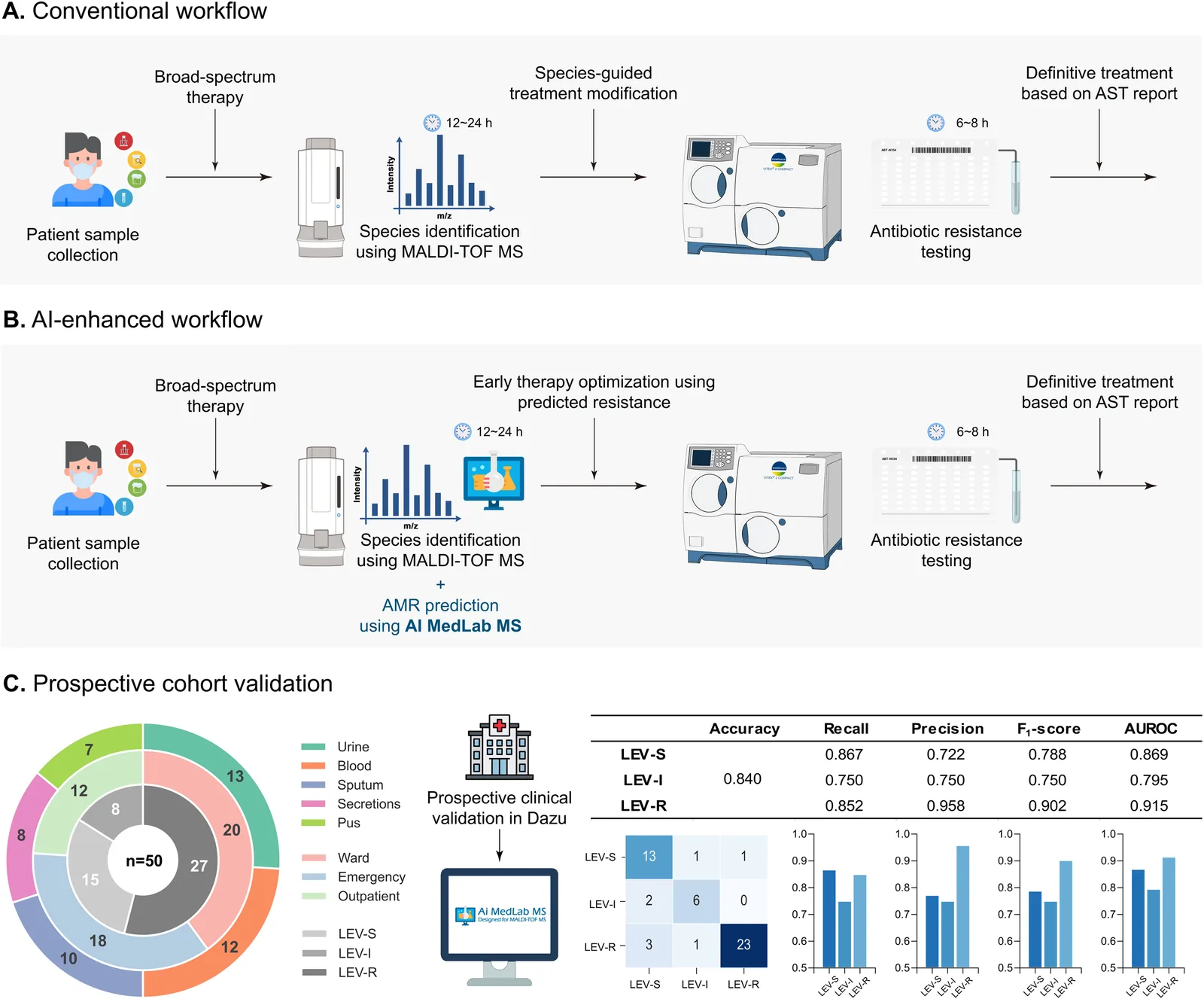
Clinical workflow comparison and prospective validation of the AI MedLab MS platform. (**A**) Conventional clinical workflow. After patient sample collection, species identification using MALDI-TOF MS takes 12–24 h, guiding an initial, species-guided treatment modification. Definitive treatment adjustments must wait for an additional 6–8 h for the results of conventional AST. (**B**) Ai-enhanced workflow. The AI MedLab MS platform is integrated directly after species identification. It provides an accurate AMR prediction within 2 h, enabling early therapy optimization 18–24 h ahead of the conventional AST report. The final AST report still serves as definitive confirmation. (**C**) Prospective cohort validation results. The platform was prospectively validated on a cohort of 50 clinical *E. coli* isolates collected from the Affiliated Dazu’s Hospital of Chongqing Medical University. Donut charts illustrate the distribution of specimen sources, hospital departments, and levofloxacin resistance phenotypes. The performance table, confusion matrix, and bar charts summarize the predictive performance of the platform in this real-world setting, achieving an overall accuracy of 84.0%**.**

To determine the platform’s utility in this accelerated workflow (Fig. 4A), which provides results within 2 h (Fig. 4B), we conducted a prospective validation on an independent cohort of 50 *E. coli* isolates collected from diverse specimen sources, including sputum, urine, and blood (Fig. 4C). Unlike the development set, these samples reflected the natural prevalence of resistance phenotypes (15 LEV-S, 8 LEV-I, and 27 LEV-R) encountered in daily practice.

In this prospective setting, the platform achieved an overall categorical agreement of 84.0% with the comparator method (VITEK 2 Compact system) across the three susceptibility categories. Categorical discrepancy analysis revealed a very major discrepancy (VMD) rate of 11.1% (3/27), a major discrepancy (MD) rate of 6.7% (1/15), and a minor discrepancy (mD) rate of 8.0% (4/50). Most critically, for clinical decision-making, the model achieved a precision of 95.8% in identifying resistant isolates.

## DISCUSSION

In this study, we established and validated AI MedLab MS, a pioneering platform that integrates an adapted relative growth method with dynamic MALDI-TOF MS and AI to decode the temporal evolution of bacterial responses to antibiotics. This integration, with AI serving as the core analytical engine, aligns with our objective of developing a rapid AST tool—specifically for determining the susceptibility of *E. coli* to LEV as outlined in the study’s experimental design. Our findings demonstrate that shifting the diagnostic paradigm from single-timepoint measurements to time-resolved trajectory analysis enables the rapid and precise prediction of the susceptibility of *E. coli* to LEV within 2 h. By capturing the fine-grained kinetic interplay between pathogens and drugs, our platform not only outperforms existing single-timepoint MALDI-TOF MS-based rapid AST methods in diagnostic accuracy but also provides a novel methodology for visualizing the dynamic behavior of bacterial populations under stress.

The central innovation of our work lies in the shift from single-timepoint measurements to dynamic, time-resolved monitoring of bacterial growth. Existing MALDI-TOF MS-based methods, such as MBT-ASTRA, rely on a single spectral snapshot taken during the transient phase of the bacterium–antibiotic interaction, whereas conventional phenotypic methods achieve reliable endpoint measurements only after 12–24 h of incubation (30). Although the existing MBT-ASTRA method has shortened this timeframe, our results indicate that it still faces challenges, particularly in the accurate classification of LEV-I strains, where we observed a precision of only 0.422 (Table S3). AI MedLab MS overcomes this limitation by implementing the RBD-RG algorithm, which captures fine-grained proteomic shifts across multiple time points. This dynamic approach translated into a substantial performance gain, improving overall accuracy by approximately 7% in internal testing compared with the MBT-ASTRA method. These findings suggest that early subtle changes in protein expression patterns, often missed by endpoint assays, contain critical information that is predictive of the final resistance phenotype.

AI MedLab MS also addresses a key limitation of conventional AST in detecting heteroresistance (HR), a phenomenon in which resistant subpopulations exist within a predominantly susceptible population and can drive therapeutic failure (31, 32). Conventional single-colony AST overlooks such intrasample heterogeneity: testing individual colonies in isolation may miss resistant subpopulations and produce misleading phenotypic assignments. AI MedLab MS circumvents this problem by analyzing the bacterial population as a whole, without colony separation, thereby providing a more accurate phenotypic readout. The platform does not directly identify HR-associated gene mutations; rather, its strength lies in capturing the collective phenotypic behavior of the population.

The clinical implications of AI MedLab MS are substantial. The platform’s ability to deliver a reliable resistance profile within 2 h directly addresses the urgent need for rapid diagnostics in managing severe infections caused by pathogens such as *E. coli*. In our prospective validation, the platform achieved a categorical agreement of 84.0% with the comparator method (VITEK 2 Compact system), with VMD, MD, and mD rates of 11.1%, 6.7%, and 8.0%, respectively. The precision for identifying resistant strains was 95.8% (23/24). This rapid turnaround creates a critical window for clinicians to de-escalate broad-spectrum antibiotics or switch to more effective targeted therapies much earlier than is currently possible. Such timely intervention is fundamental for improving patient outcomes, reducing the selective pressure that drives resistance, and supporting hospital-wide antimicrobial stewardship programs (33, 34). The successful deployment of the platform as a web-based clinical decision support system further underscores its readiness for integration into modern clinical microbiology workflows.

Despite these promising results, several limitations should be noted. First, the models were developed and validated using a moderate number of clinical isolates from a single geographic region, which may limit the generalizability of our findings (35, 36). The prospective validation cohort, although providing real-world evidence, was limited in size. Second, this study examined only one pathogen–drug combination (*E. coli* and LEV). Whether the dynamic signatures identified here are unique to fluoroquinolones or reflect a broader bacterial stress response remains to be determined across additional antibiotic classes. Finally, although classification of intermediate strains improved, it remains the most challenging category, as the prospective validation results indicate.

Consistent with standardized evaluation guidelines, we report categorical agreement as the primary performance indicator. In the binary classification analysis (resistant vs non-resistant), categorical agreement reached 90.0%, meeting the routine clinical testing standard of ≥90%. The three-class categorical agreement (LEV-S/LEV-I/LEV-R) was 84.0%. This gap was primarily driven by intermediate isolates, which often produce ambiguous spectral signals under short incubation times. Notably, three of the four binary VMDs arose from LEV-I misclassification, underscoring the challenge posed by this phenotype. Larger cohorts will be needed to improve three-class performance and reduce VMD rates.

The categorical discrepancy rates require contextual interpretation. The VMD rate of 11.1% exceeds the ≤3% CLSI benchmark, indicating that some truly resistant isolates were misclassified as susceptible. This is the most clinically consequential error type, as it could lead to the prescription of an ineffective antibiotic. However, AI MedLab MS is designed as a rapid preliminary screening tool to complement, not replace, conventional AST. In current clinical practice, definitive treatment decisions remain guided by standard AST results, which serve as the confirmatory reference. The platform’s value lies in providing actionable resistance information within 2 h, enabling early therapeutic adjustment while awaiting confirmatory testing. Larger training cohorts, automated workflows, and refined classification thresholds are expected to reduce discrepancy rates.

Several technical aspects also warrant discussion. LRP analysis offers valuable insight into the model’s decision-making process; however, it cannot substitute for proteomic and genomic studies needed to identify the specific proteins and genetic mechanisms underlying the observed spectral changes. Moreover, the current workflow depends on substantial manual intervention, including multi-timepoint sampling, centrifugation, matrix application, and target spotting. Although AST results are available within 2 h, these hands-on steps constrain throughput in high-volume clinical settings. Future development should therefore prioritize automation—through microfluidic sample processing, optimized DOT-MGA designs, and robotic liquid handling—to transition the platform from a research tool toward clinical deployment.

Despite these limitations, our findings highlight the considerable potential of dynamic proteomic trajectories for AMR detection, particularly for challenging phenotypes such as HR. Future studies should extend the platform to additional clinically relevant pathogens and antimicrobial classes. Integrating dynamic mass spectral data with genomic and proteomic analyses may further elucidate the molecular mechanisms underlying HR. Ultimately, large-scale, multicenter prospective trials will be required to validate the platform’s clinical utility and define its role in personalized infectious disease management.

In conclusion, this study demonstrates that the AI MedLab MS platform, by leveraging dynamic proteomic fingerprinting, can address the key limitations of conventional antimicrobial susceptibility testing. By capturing the temporal changes in bacterial responses to antimicrobials, the platform enables rapid, accurate, and interpretable classification of antimicrobial resistance, highlighting the potential of dynamic trajectory analysis as a new paradigm in rapid diagnostics.

## Data Availability

All data generated or analyzed during this study are included in this published article.

## SUPPLEMENTAL MATERIAL

Fig. S1 to S7; Tables S1 to S4 spectrum.01035-26-s0001.docx Fig. S1: Detailed clinical cohort composition and inclusion criteria. Fig. S2: Step-by-step protocol for sample preparation and spectral acquisition. Fig. S3: Computational pipeline for spectral preprocessing and RBD-RG matrix construction. Fig. S4: Correction of inter-instrument spectral variation via automated m/z alignment. Fig. S5: Definition and quantitative characterization of the RNase B reference signature for automated QC. Fig. S6: The automated workflow for MALDI-TOF MS spectral quality control and its validation. Fig. S7: Automated data processing in the AI MedLab MS platform. Table S1: Statistical characterization of the five reference peaks of RNase B. Table S2: Strategy for random grid search to identify optimal hyperparameters. Table S3: Performance benchmarking against the MBT-ASTRA method. Table S4: Comparative performance of classification models based on the RBD-RG method.

ASM does not own the copyrights to Supplemental Material that may be linked to, or accessed through, an article. The authors have granted ASM a non-exclusive, world-wide license to publish the Supplemental Material files. Please contact the corresponding author directly for reuse.
