# Supplementary material for: Evaluation of an adapted relative growth method by MALDI-TOF MS for rapid determination of the susceptibility of Escherichia coli to levofloxacin
Source: Microbiol Spectr. 2026 Aug 4;14(9):e01035-26. doi: 10.1128/spectrum.01035-26 (PMC13532333; doi:10.1128/spectrum.01035-26)
Supplement: Supplementary file 1 [file spectrum.01035-26-s0001.docx]

**Evaluation of an adapted relative growth method by MALDI-TOF MS for rapid determination of the susceptibility of *Escherichia coli* to levofloxacin**

Yulong Liu^1, 2^, Niqi Xie^1^, Weiwei Hu^1^, Xiaoqin Zeng^1^, Siying Sun^1^, Lijuan Pan^1^, Hongyou Chen^1^, Xuelian Peng^1^, Chunyan Yang^1^, Baoru Han^1, 2*^, Jin Li^1*^

*^1^ Department of Laboratory Medicine, The Affiliated Dazu’s Hospital of Chongqing Medical University, Chongqing, China;*

*^2^ College of Artificial Intelligence Medicine, Chongqing Medical University, Chongqing, China.*

^*^Co-corresponding authors:

Jin Li Email: [lijin@hospital.cqmu.edu.cn](mailto:lijin@hospital.cqmu.edu.cn);

Baoru Han Email: [baoruhan@cqmu.edu.cn](mailto:baoruhan@cqmu.edu.cn);

Yulong Liu, Niqi Xie, and Weiwei Hu contributed equally to this aridle.

**Fig. S1 Detailed clinical cohort composition and inclusion criteria.**

**(A) Distribution of *E. coli* isolates across four medical centers in Chongqing.** The bar charts illustrate the sample size and distribution of levofloxacin-susceptible (LEV-S), -intermediate (LEV-I), and -resistant (LEV-R) phenotypes. Data are stratified by specimen source (predominantly urine, sputum, and blood) and patient admission origin (ward, emergency, outpatient), reflecting the diversity of the clinical cohort.

**(B) Workflow for bacterial identification and reference AST.** Clinical isolates were subcultured on Columbia blood agar to obtain pure colonies. Species identification was performed using MALDI-TOF MS; only isolates achieving a confidence score ≥2.0 were included in the study to ensure taxonomic accuracy. Reference AST was conducted using the VITEK 2 Compact system (card AST-N334) to determine LEV phenotypes in strict accordance with CLSI guidelines.

**Fig. S2 Step-by-step protocol for sample preparation and spectral acquisition.**

Standardized bacterial suspensions (0.5 McFarland) are incubated in parallel with or without levofloxacin, with aliquots (500 µL) harvested sequentially at 30-minute intervals over 120 minutes. Harvested samples undergo centrifugation and an optimized ethanol-formic acid extraction step (washing with sterile water, followed by 75% ethanol and 70% FA treatment) to maximize protein yield and spectral quality. Crucially, RNase B (internal standard, 20 g/L) is dissolved in 100% acetonitrile (ACN) and added during the acetonitrile extraction step to ensure quantitative consistency during spectral acquisition. Finally, spectra are acquired in linear positive mode, generating spectral plots that visualize the kinetic evolution of bacterial proteomic profiles (*E.coli* + LEV vs. LEV) over the time course.

 **Fig. S3 Computational pipeline for spectral preprocessing and RBD-RG matrix construction.**

The flowchart details the data processing logic from raw signals to AI-ready features. Raw mass spectra first undergo baseline subtraction and smoothing to reduce instrumental noise. Calibration and alignment are then anchored to the characteristic RNase B internal standard peak (highlighted by the red arrow, theoretical m/z at ~15064 for [M+H]^+^) to correct for inter-spot mass drift. Following peak detection, an automated quality control (QC) mechanism evaluates spectra based on the signal-to-noise ratio and morphology of the internal standard; "Qualified" spectra with distinct IS peaks are retained, while "Unqualified" spectra are automatically filtered out. High-quality spectra are ultimately transformed into the structured RBD-RG matrix, which quantifies relative growth across four time points and seven m/z sub-intervals for model input.

**Fig. S4 Correction of inter-instrument spectral variation via automated m/z alignment.**

**(A)** Raw spectra of quality control (QC) samples exhibiting significant m/z drift due to analysis on different instruments prior to alignment.

**(B)** Following automated alignment, spectral consistency was restored by anchoring a characteristic peak of the internal standard protein (in this case, the second peak of RNase B) to a reference value of 15064 m/z.

**Fig. S5 Definition and quantitative characterization of the RNase B reference signature for automated QC.**

**(A)** A representative mass spectrum of the internal standard, RNase B, used to establish the reference standard. The relevant m/z range is partitioned into five distinct sub-intervals (labeled I–V), each designed to capture a characteristic protein peak. The red cross marks the apex of the identified characteristic peak within each interval.

**(B)** Box plots illustrating the statistical distribution of the maximum peak intensity for the characteristic protein peak within each of the five defined sub-intervals. These distributions were derived from a curated dataset of reference RNase B spectra, thereby establishing the expected quantitative signature for a passing QC sample.

**Fig. S6 The automated workflow for MALDI-TOF MS spectral quality control and its validation.**

The top panel provides a schematic of the automated QC pipeline. This process involves extracting features specific to an internal standard (RNase B) from raw MS spectra and generating a numerical quality score via statistical comparison to a high-quality reference. Spectra are then accepted (QC Pass, Score ≥ 60) or rejected (QC Fail, Score < 60) based on this score. The lower panels validate the workflow's performance using representative examples.

**(A)** High-quality spectra, characterized by a well-defined and high-intensity RNase B peak signature, consistently receive high scores (e.g., ≥ 85) and thereby pass the QC assessment.

**(B)** In contrast, low-quality spectra, which are dominated by noise and lack a discernible RNase B signal, are correctly assigned a score of zero and are consequently filtered out by the workflow.

**Fig. S7 Automated data processing in the AI MedLab MS platform.**

**(A)** Upon patient selection, the Data Analysis Workstation launches and automatically imports the associated mass spectrometry data for processing.

**(B)** An automated pipeline executes feature peak detection, spectral quality control, and dynamic relative growth measurement. Processed spectra from all four time points are displayed, with antibiotic-treated and control samples distinguished by color coding for direct comparison.
**Table S1 Statistical characterization of the five reference peaks of RNase B.**

The table lists the mean, standard deviation (std), and confidence intervals (defined by 1σ, 2σ, and 3σ) for both the peak intensity and m/z value of each of the five peaks.

| **Variables** | **mean** | **std** | **Excellent** | **Good** | **Pass** |
| --- | --- | --- | --- | --- | --- |
| Int_RNase B_ **_I_** | 1066.443 | 47.823 | (1018.620, 1114.266) | (970.797, 1162.089) | (922.974, 1209.912) |
| Int_RNase B_ **_II_** | 997.418 | 2.237 | (995.181, 999.655) | (992.944, 1001.892) | (990.707, 1004.129) |
| Int_RNase B_ **_III_** | 159.015 | 22.999 | (136.016, 182.014) | (113.017, 205.013) | (90.018, 228.012) |
| Int_RNase B_ **_IV_** | 425.878 | 48.05 | (377.828, 473.928) | (329.778, 521.978) | (281.728, 570.028) |
| Int_RNase B_ **_V_** | 164.325 | 41.765 | (122.560, 206.090) | (80.796, 247.854) | (39.031, 289.619) |
| m/z_RNase B_ **_I_** | 14901.351 | 0.866 | (14900.485, 14902.217) | (14899.618, 14903.084) | (14898.752, 14903.950) |
| m/z_RNase B_ **_II_** | 15062.747 | 0.793 | (15061.954, 15063.540) | (15061.161, 15064.333) | (15060.368, 15065.126) |
| m/z_RNase B_ **_III_** | 15223.78 | 1.379 | (15222.401, 15225.159) | (15221.023, 15226.537) | (15219.644, 15227.916) |
| m/z_RNase B_ **_IV_** | 15388.85 | 1.057 | (15387.793, 15389.907) | (15386.736, 15390.964) | (15385.679, 15392.021) |
| m/z_RNase B_ **_V_** | 15548.711 | 1.449 | (15547.262, 15550.160) | (15545.814, 15551.608) | (15544.365, 15553.057) |

**Table S2 Strategy for random grid search to identify optimal hyperparameters.**

| **Model** | **API** | **Parameters** | |
| --- | --- | --- | --- |
| LR | LogisticRegression () | C | [0.01, 0.1, 1, **10**, 100] |
|  |  | penalty | [**"l1"**, "l2"] |
|  |  | solver | [**"liblinear"**, "saga"] |
| DT | DecisionTreeClassifier () | criterion | ["gini", **"entropy"**] |
|  |  | splitter | ["best"] |
|  |  | max_depth | [**None**, 10, 15, 20] |
|  |  | min_samples_split | [10, **20**, 30] |
|  |  | min_samples_leaf | [5, 10, **15**] |
|  |  | max_features | ["sqrt", **"log2"**] |
| XGB | XGBClassifier () | learning_rate | [0.001, 0.01, 0.1, 0.2, 0.3, **0.5**] |
|  |  | n_estimators | [100, 200, 500, **1000**] |
| MLP | MLPClassifier () | hidden_layer_sizes | [(50,), (100,), **(50, 50)**, (500,),  (500, 500), (1000,), (1000, 1000)] |
|  |  | activation | ["tanh", "relu"] |
|  |  | solver | ["sgd", **"adam"**] |
|  |  | alpha | [0.0001, **0.001**] |
|  |  | learning_rate | ["constant", **"adaptive"**] |
|  |  | max_iter | [200, **500**] |

**Table S3 Performance benchmarking against the MBT-ASTRA method.**

| **Methods** | | **Cut-off** | **Accuracy** | **Precision** | **Recall** | **F_1__score** | **AUROC** |
| --- | --- | --- | --- | --- | --- | --- | --- |
| **MBT-ASTAR** | LEV-S | ≤ 0.101 | 0.876 | 0.998 | 0.865 | 0.927 | - |
|  | LEV-I | 0.101-0.396 |  | 0.422 | 0.986 | 0.591 |  |
|  | LEV-R | ≥ 0.396 |  | 1.000 | 0.864 | 0.927 |  |

**Table S4 Comparative performance of classification models based on the RBD-RG method.**

|  | **Models** | **AST** | **Accuracy** | **Recall** | **Precision** | **F_1_-score** | **AUROC** |
| --- | --- | --- | --- | --- | --- | --- | --- |
| **Internal testing** | LR | LEV-S | 0.926 ± 0.034 | 0.928 ± 0.109 | 0.898 ± 0.080 | 0.906 ± 0.061 | 0.970 ± 0.045 |
|  |  | LEV-I |  | 0.940 ± 0.087 | 0.975 ± 0.038 | 0.954 ± 0.045 | 0.993 ± 0.011 |
|  |  | LEV-R |  | 0.910 ± 0.084 | 0.932 ± 0.088 | 0.916 ± 0.056 | 0.989 ± 0.011 |
|  | DT | LEV-S | 0.733 ± 0.061 | 0.668 ± 0.188 | 0.700 ± 0.092 | 0.666 ± 0.109 | 0.776 ± 0.059 |
|  |  | LEV-I |  | 0.723 ± 0.161 | 0.679 ± 0.102 | 0.679 ± 0.067 | 0.774 ± 0.062 |
|  |  | LEV-R |  | 0.808 ± 0.206 | 0.894 ± 0.121 | 0.831 ± 0.154 | 0.881 ± 0.107 |
|  | XGB | LEV-S | 0.893 ± 0.073 | 0.948 ± 0.084 | 0.910 ± 0.084 | 0.923 ± 0.055 | 0.985 ± 0.035 |
|  |  | LEV-I |  | 0.850 ± 0.120 | 0.919 ± 0.110 | 0.877 ± 0.094 | 0.970 ± 0.040 |
|  |  | LEV-R |  | 0.880 ± 0.119 | 0.892 ± 0.127 | 0.878 ± 0.093 | 0.979 ± 0.028 |
|  | MLP | LEV-S | 0.944 ± 0.040 | 0.925 ± 0.110 | 0.930 ± 0.066 | 0.923 ± 0.066 | 0.982 ± 0.020 |
|  |  | LEV-I |  | 0.953 ± 0.056 | 0.986 ± 0.018 | 0.968 ± 0.029 | 0.993 ± 0.005 |
|  |  | LEV-R |  | 0.953 ± 0.042 | 0.934 ± 0.091 | 0.940 ± 0.049 | 0.982 ± 0.019 |
| **External validation** | LR | LEV-S | 0.873 ± 0.052 | 0.830 ± 0.083 | 0.997 ± 0.008 | 0.904 ± 0.050 | 0.997 ± 0.006 |
|  |  | LEV-I |  | 0.859 ± 0.078 | 0.952 ± 0.022 | 0.901 ± 0.043 | 0.961 ± 0.026 |
|  |  | LEV-R |  | 1.000 ± 0.000 | 0.623 ± 0.175 | 0.754 ± 0.121 | 1.000 ± 0.001 |
|  | DT | LEV-S | 0.734 ± 0.094 | 0.577 ± 0.190 | 0.444 ± 0.177 | 0.493 ± 0.170 | 0.732 ± 0.131 |
|  |  | LEV-I |  | 0.723 ± 0.134 | 0.891 ± 0.044 | 0.792 ± 0.089 | 0.797 ± 0.089 |
|  |  | LEV-R |  | 1.000 ± 0.000 | 0.754 ± 0.197 | 0.844 ± 0.143 | 0.974 ± 0.030 |
|  | XGB | LEV-S | 0.859 ± 0.107 | 0.795 ± 0.066 | 0.732 ± 0.155 | 0.756 ± 0.119 | 0.929 ± 0.039 |
|  |  | LEV-I |  | 0.848 ± 0.144 | 0.945 ± 0.010 | 0.887 ± 0.094 | 0.951 ± 0.014 |
|  |  | LEV-R |  | 1.000 ± 0.000 | 0.807 ± 0.187 | 0.880 ± 0.130 | 1.000 ± 0.000 |
|  | MLP | LEV-S | 0.967 ± 0.018 | 0.845 ± 0.095 | 0.996 ± 0.007 | 0.911 ± 0.057 | 0.940 ± 0.058 |
|  |  | LEV-I |  | 0.992 ± 0.007 | 0.972 ± 0.016 | 0.982 ± 0.010 | 0.984 ± 0.014 |
|  |  | LEV-R |  | 1.000 ± 0.000 | 0.916 ± 0.063 | 0.955 ± 0.035 | 0.995 ± 0.006 |
